# Enacting Space in Virtual Reality: A Comparison Between Money’s Road Map Test and Its Virtual Version

**DOI:** 10.3389/fpsyg.2018.02410

**Published:** 2018-12-05

**Authors:** Francesca Morganti

**Affiliations:** Department of Human and Social Sciences, University of Bergamo, Bergamo, Italy

**Keywords:** enactive cognition, spatial cognition, virtual reality, Money’s Road Map test, egocentric and allocentric coordinates

## Abstract

In the field of spatial cognition research the mutual relationship between perception and action that brings out spatial orientation was lately investigated. Besides, the sameness between creating a cognitive map from the exploration of a not simulated environment, from the use of an allocentric (survey-like) sketched map, and from the interaction with egocentric (route-like) 3D virtual environments, is generally contrived. To understand if different embodied affordances could provide different knowledge organization during wayfinding through the use of distinct spatial simulations, the same group of 61 healthy subjects experienced both the classical version of the Money’s Road Map test (M-RMT) and a virtual reality version of the Road Map test (VR-RMT). The M-RMT requires a allocentric to egocentric right/left reasoning to explore a stylized city provided in a survey perspective. The VR-RMT is a 3D version of the same environment through which participants can actively navigate by choosing egocentric-based right/left directions in a route perspective. The results showed that the different embodiments afforded by the two environments and the increasing complexity in turn types provides different spatial outcomes. Results were discussed according to the sensorimotor coupling theory provided from the enactive cognition approach and significances for spatial cognition research were provided.

## Introduction

The rearmost neuroscientific findings have implied a large overlaying between action and perception inserting the challenge of a spatial cognition research within the enactive approach. This cognitive framework change requires the reshaping of what “interaction” means (Morganti, 2016).

Within the embodied cognition perspective, in fact, is the sensorimotor coupling of the agent’s action and of her environmental perception that shapes the possibilities for spatial exploration (Gibson, 1979; Varela et al., 1991; Thompson and Varela, 2001). Thus, spatial cognition derives from the agent’s management of an action and from the maintenance of her moment-by-moment sensorimotor schema. This schema “guides” the agent in how to appropriately execute her movements in the specific situation in which she finds herself and what sorts of feedback to expect from the environment (Carassa et al., 2005).

The enactive approach on interaction has some unequivocal implications for spatial cognition research. Orientation, in fact, is a high level cognitive ability that comprises the construction and use of a spatial representation of the context within which an action is performed. To be effectual it exacts information originated from multiple domains, the perpetual placement of the individual who is acting, combined with the planning of behaviors that are claimed to be ranged with the agent (Gramann et al., 2005). Through catching the opportunities for actions during a new environment exploration, the agent organizes spatial knowledge through egocentred maps (derived from routes traveled in which borders and landmarks can be individuated) and, in the meanwhile, to place herself in the environment by using allocentred maps (based on survey pathway combination) (Brunyeì et al., 2012). Together route and survey viewpoints can be contemplated as “commonplace.” Moreover, their reciprocal conversions are an essential procedures backing a productive navigation of intricate environments (Hartley et al., 2003; Ishikawa and Montello, 2006).

Therefore, wayfinding can be conceived as based on the continuous equilibrium between egocentric and allocentric perspectives during the agent’s perception–action coupling. Thus, the allocentric perspective supports spatial understandings while the agent is involved in a wayfinding that provides her with egocentric information. Nevertheless, by underwriting what surrounding dynamics are the most befitting among the numerous available at the time, an agent has possibility to plan in advance a path, even in a partly unknown environment, by creating spatial inferences (Morganti et al., 2007). Spatial plans, in fact, can’t be considered as pure allocentric action representations (that have to be followed thoughtlessly), but they turn out to be controllers for action to be additionally detailed in the egocentric-based interaction with the surrounding space.

Neuroscience studies support this allocentric/egocentric balance for spatial cognition (Serino et al., 2014), benehating the role of the retrosplenial cortex in the merging of the allocentric data (provided by the Papez circuit) with the egocentric ones received from parietal areas (Burgess, 2006). These neuroscientific evidences evoke how the spatial orientation is inseparable from the embodied perspective and from the specific opportunities for action caught in the explored context (Gunzelmann, 2008).

Thus it is possible to assume that, during a new environment exploration, an agent bodily enacts with a context in a continuous developing process. Accordingly, exploration can be considered as not simply guided by agent goals or motor actions, rather from the everlasting “hook up” of perceptions and actions that creates the agent’s way of experiencing the context in which she is included. Moreover, when an agent and a specific environment interact, they are structurally coupled and they co-emerge.

In the last decade, due to the progression of technology, virtual reality simulations were widely introduced in neuroscience and experimental psychology (Morganti, 2004). Together with paper and pencil simulation of environments (such as building plans, city maps, and so on) they were largely used to study spatial cognition. Both these kind of simulations have been generally considered as equivalent to natural place explorations. Moreover, virtual reality by the use of motion devices (such as head- and limb-trackers) can provide a configuration of “natural-like” sensorimotor coupling within the digital environment, providing the agent with the possibility of actively catch opportunities for action in a computerized three-dimensional space. Even if the spatial knowledge organization derived from virtual environment simulation can be linked to an embodied perception grounded on a situated action, a research question arises here: might the coupling between an agent and the perceptive data provided by the digital environment create a different kind of spatial knowledge representation from the one obtainable to classical map-use? Might it have an impact on actions’ choice that an explorer can perform within the environment? Adopting the enactive perspective to spatial simulation-based interaction, in fact, requires reconsidering the definition of the nature of the coupling between the agent and the context and of the possible reciprocal modifications and changes between them (Mellet-d’Huart, 2006). Map-based and virtual reality simulated contexts can provide an agent with specific affordances, and with the possibility to obtain spatial representations from a peculiar coupling with a device-mediated sensorimotor system. This could result in form of agent-environment regularities (e.g., spatial invariants) different in virtual reality simulated and map-based spaces understanding. We consider that as the main issue of our research.

To study how the agent-environment coupling could be in two different spatial simulations, the same group of healthy subjects experienced both the classical paper version of the Money’s Road Map test (M-RMT – Money et al., 1967) and a virtual reality version of the Road Map test (VR-RMT – Morganti et al., 2009). As it includes the allocentric egocentric coordination and it is considered an ecologically-like spatial simulation, in the neuropsychological evaluation of spatial ability after brain injury the classical version of M-RMT is generally included. To be solved, in fact, this task requires to egocentrically think about a right/left rotation during the exploration of a sketched city map provided in the allocentric perspective. As the other side of the medal the nowadays exist a virtual VR-RMT that provides participants with an explorable three-dimensional version of the M-RMT in which there is the possibility to actively choose the right/left turns from a egocentric perspective.

The main aim of research is to compare the M-RMT and the VR-RMT in order to understand whether there is any difference between set-out a right/left turn on a body axis (as in the M-RMT) and performing it (as in the VR-RMT) in order to obtain a spatial perspective from the simulated world. Accordingly, our research methodology requires the following steps from participants:

- In the M-RMT condition, participants first look at the map, then delineate how to move on the body axis and finally obtain (and have to keep in mind) the spatial perspective derived from the turn.
- In the VR-RMT condition, participants first look at the map, then in the virtual environment can actively turn right or left on the body axis and obtain the spatial perspective accordingly.

The comparison between M-RMT and VR-RMT proposed here introduces two different spatial simulations that might provide participants with different embodied affordances. They can, in fact, be considered as tightly linked with different sensory-motor coupling situations. In particular, in the VR-MRT an agent is required to plan in advance a right/left turn and to continuously create relationships between the perspectives obtained in the environment with the result of each turns. While in the M-RMT the agent has to translate information perceived on a map to a possibility of action that can be performed in the environment (but only imagined and taken in mind during exploration). Thus, it is possible to hypothesize that to observe the resulting of a right/left turn in the virtual environment requires unalike cognitive efforts than to ground it on a pure internal cognitive process as in the M-RMT process. These differences in the sensorimotor coupling between the perceptual information and the turn possibilities on the VR-RMT and M-RMT involves a different idea of body (device mediated and not-mediated ones) and it might create different experience for the agent during navigation. Moreover, the invariants of the physical world, obtainable from the active interaction within the virtual environment (the peculiar spatial perspectives faced after a right/left turn in the VR-RMT) might guide the agent’s wayfinding in a different manner from the ones provided by the necessity inference on how a spatial perspective can be following a right/left turn in the M-RMT.

Accordingly, it is hypothesized here that the non-identical activities performed in the differently simulated environments will result in distinguishable orientation outcomes. Thus, the main hypothesis is that the peculiar M-RMT and VR-MRT sensorimotor coupling can have role in performing wayfinding and also in facing the increasing complexity of the right/left turns during exploration. Finally, we would like to understand whether, only for the VR-RMT, some individual differences exist in spatial orientation derived from age and computer interaction expertise. We expect that the rotation in VR can be difficult to perform if the participant does not have sufficient expertise in managing computer-based simulations or might present a slight cognitive frailty due to their specific age cohort.

## Materials and Methods

### Materials

**FIGURE 1:**
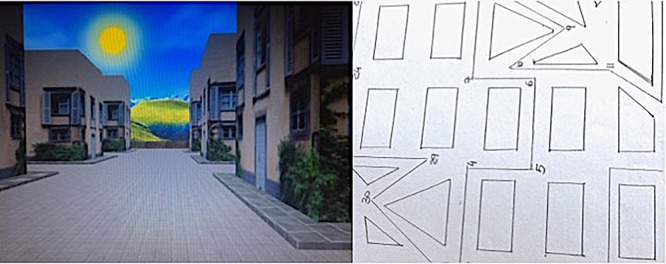
Snapshots of VR-RMT and M-RMT used in the study.

The M-RMT (Money et al., 1967) is a test of left–right discrimination. It consists of a stylized city map, depicted in Figure 1, in which participants indicate on a 32-step dotted pathway the direction taken at each turn (left or right) in order to follow a designated route. The answers require an allocentric to egocentric based reasoning, because the dotted pathway follows an erratic trace both away from and toward the agent, who is not allowed to turn the map or to make head and body movements to give the correct answer.

The VR-RMT (Morganti et al., 2009), is a virtual reality version of the M-RMT, in which the paper and pencil version is turned into an actively navigable city from an egocentric perspective. No landmarks are depicted as navigation cues, and all the buildings in the virtual simulation have the same texture. The VR-RMT was developed with 3D Game Studio software by which 3D buildings were developed on the basis of buildings’ shape and position in the paper and pencil version of the test. The navigation speed was constant. It was approximately 5 m and 40° per second.

The VR-RMT was administered on an Intel personal computer and was presented on a wall by a video projector that provides a 1,50 m × 1 m image. The participants was seated in a chair approximately 2 m from the virtual environment image depicted on the wall and moved in the virtual environment using a facilitate narrow keyboard (The QueenKey 2.5 × 2.5 narrow keyboard) placed on a small table in front of them.

A snapshot of the M-RMT and VR-RMT was provided in Figure 1.

### Participants

In this study, we administered both the M-RMT and the VR-RMT to 83 healthy right-handed volunteers aged from 30 to 80 years. Sixty one participants remains enrolled in the study after the assessment of keyboard use and virtual reality familiarity whose mean age was 56.82 and *SD* = 15.47. We divided participants into three groups according to their age. The experimental population presents 19 Young Adults (YA, from 30 to 49 years old), 19 Adults (A, from 49 to 64 years old), and 23 Old Adults (OA, from 65 to 80 years old). In order to avoid confounding variables, such as sex differences in spatial skills, male and female gender was balanced. The participants included 31 females and 30 males with 5 to 19 years of education (Mean = 12,08; *SD* = 3,62). All subjects participated as volunteers and gave informed consent for their data treatment. No participant had a clinical history of neurological and mood disorders such as anxiety/depression.

### Procedure

In order to exclude participants with deficits in cognitive domains, the Mini Mental State Examination (MMSE – Folstein et al., 1975) was performed. Participants who had a poor performance (cut off value 24/30) on the MMSE were excluded. After the cognitive evaluation, participants were introduced to the experimental phase.

Using a different virtual environment from the experimental one, a 10-min training session was run to familiarize the participants with the use of a keyboard for navigating in virtual reality. After 10 min, if participants felt comfortable with the keyboard and had satisfactorily demonstrated their ability to guide themselves within the environment, the participants were included in the experimental study. If the participant was not able to navigate the training virtual environment, she was excluded from participation in the experiment. The participants included in the study were also evaluated as slight/average/good in computer interaction by the experimenter, according to the expertise they showed in managing the narrow keyboard to move in the virtual environment. If, according to three expert observers, they were able to quickly move in the keyboard and understanding the correspondence between their finger movements and the effect of them in the virtual environment, they were classified as good. If they require some more training they were classified as average, if they ask for some support from the experimenter they were classified as slight. Nevertheless, all the participant at the end of the training session have to perform the task without experimenter help to be enrolled in the study.

In the experimental phase, the participants were tested individually. They were asked to perform both the M-RMT and the VR-RMT. The two versions of the test were randomly presented to participants. Half the participants performed the M-RMT first and the other half of the participants performed the VR-RMT first. In both the version of the tests, the starting point and the target point were clearly indicated.

In the M-RMT, we asked participants to follow on the sketched map a route taken by a hypothetical traveler. The participant was seated facing the examiner. She was asked to imagine herself moving along a 32-turn (choice points) route indicated by the experimenter on the map. Then, she had to spatially rotate himself to ascertain whether a right or left turn was demanded at each multiple-choice intersection. At each turn point, the participant had to answer the examiner’s question: “In order to follow the depicted route, at this point would you be turning right or left?” The map always remained in a fixed position in front of the subjects, who were not allowed to alter their position to facilitate right–left judgments.

In the VR-RMT, the participants viewed virtual environment depicted on the screen with the paper version of the test placed in the table in front of them. While the examiner followed with her finger the route indicated by a dotted line on the paper version of the test, the participant decided which direction she must turn in the virtual environment and turned at each of 32 intersections.

In the M-RMT condition at the top side of the paper the north direction can be easily visible. In the VR-RMT, a sun straight visible from the participant’s starting point indicated the corresponding north direction. Before the start of the VR-RMT exploration, the correspondence between the starting position on the paper and in the virtual environment was clearly indicated to participants. Participants could see the paper version of the test during VR-RMT navigation, but they can’t rotate the paper in order to follow to the direction taken in the virtual environment. Participants could use the north-sun correspondence to re-orientate themselves during the virtual exploration. Each time the participant considered one of the 32 turn points she had reached, she had to orally relate her decision to the experimenter.

In both the M-RMT and VR-RMT, there were equal numbers of right and left turns. A 10-min time limit was imposed for completing the test.

## Results

In the first global analyses of performance, both for M-RMT and VR-RMT one point was given for a correct answer—the correct direction (right or left) at each turn—for a maximum of 32 points for each test. In order to test environment consistency first we had a positive correlation between the M-RMT and the VR_MRT (Pearson’s *r* = 0.58; *p* < 0.001).

In order to analyze the differences in exploring the two versions of the same environment a repeated measure 2x2x3 ANOVA was conducted. The statistic model includes as within factor Environment (2 levels: M-RMT/VR-MRT) ^∗^ Presentation Order (2 levels: M-RMT first/VR-MRT first) ^∗^ Age Group (3 levels: YA/A/OA) as between factors. Descriptive data are depicted in Table 1.

**Table 1:** Participant’s performances at the M-RMT and VR-RMT according to presentation order and age groups.

	Task order	Age group	Mean	*SD*	Participants
**M_RMT**	1 M-RMT first	YA (from 30 to 49)	30,18	2,040	11
		A (from 50 to 64)	28,00	3,521	6
		OA (from 65 to 80)	24,67	4,670	15
		**Total**	**27,19**	**4,425**	**32**
	2 VR-RMT first	YA (from 30 to 49)	31,13	1,126	8
		A (from 50 to 64)	27,69	3,276	13
		OA (from 65 to 80)	21,75	4,862	8
		**Total**	**27,00**	**4,877**	**29**
	Total	YA (from 30 to 49)	30,58	1,742	19
		A (from 50 to 64)	27,79	3,259	19
		OA (from 65 to 80)	23,65	4,839	23
		**Total**	**27,10**	**4,607**	**61**
**VR_RMT**	1 M-RMT first	YA (from 30 to 49)	20,00	8,050	11
		A (from 50 to 64)	11,17	7,387	6
		OA (from 65 to 80)	6,60	3,269	15
		**Total**	**12,06**	**8,455**	**32**
	2 VR-RMT first	YA (from 30 to 49)	19,25	9,377	8
		A (from 50 to 64)	7,92	3,499	13
		OA (from 65 to 80)	6,12	2,800	8
		**Total**	**10,55**	**7,721**	**29**
	Total	YA (from 30 to 49)	19,68	8,387	19
		A (from 50 to 64)	8,95	5,071	19
		OA (from 65 to 80)	6,43	3,057	23
		**Total**	**11,34**	**8,083**	**61**


Results showed a significant difference [*F*(399.21), *p* < 0.001] for the factor Environment. Participants better performed the spatial task in the M-RMT (Mean = 27.10; *SD* = 4.6) than in the VR-MRT (Mean = 11.34; *SD* = 8.08). Moreover, there is a significant difference in the interaction between Environment and Age Group [*F*(8.164), *p* < 0.001]. *Post hoc* analysis with Bonferroni adjustment revealed significant differences between YA, A, and OA. When it comes to the M-RMT, there is a better performance by the YA (*p* < 0.001) and the A (*p* < 0.001) compared to the OA; there are no significant differences between the YA and A. As far as the VR-MRT is concerned there is a better performance by the YA compared to the A (*p* < 0.001) and to the OA (*p* < 0.001); there are no significant differences between A and YA.

**FIGURE 2:**
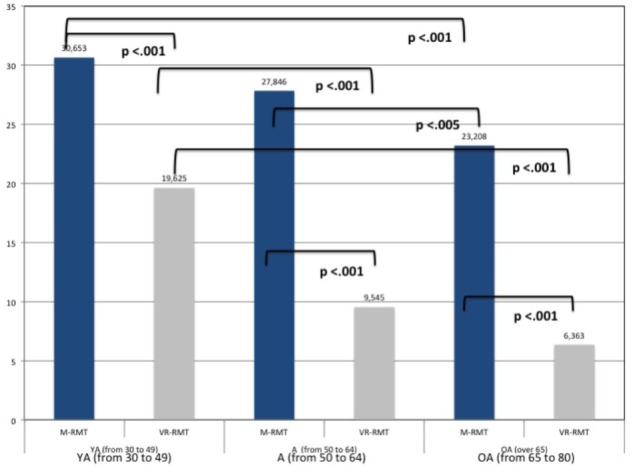
Differences in M_RMT and VR-RMT for age groups.

Moreover, pairwise means comparison (*t*-test) revealed that there are significant differences between the Environments for all the three Age Groups. Data are depicted in Figure 2.

Finally there was no significant difference in Presentation Order [*F*(1.133), *p* = 0.292], nor in Environment ^∗^ Presentation Order [*F*(0.224), *p* = 0.638], nor in the Environment ^∗^ Presentation Order ^∗^ Age Group [*F*(1.16), *p* = 0.321].

From the literature, we know that the spatial task in the M-RMT involves different levels of difficulty, defined by the direction of the virtual traveler on the map as seen from the subject’s position (Vingerhoets et al., 1996; Rainville et al., 2002).

In order to account for the fact that left–right discrimination and mental rotation are two different abilities involved in the Road Map spatial task, both for M-RMT and VR-RMT the 32 turns were divided into three types according to the differentiation described by Vingerhoets et al. (1996). As indicated by Vingerhoets and colleagues, we classified the 32 turns of the tests, placing each turn in one of the three following categories:

(a) the correct left–right turn doesn’t require mental rotation [no rotation (NR)];
(b) the correct left–right turn requires a 90° mental rotation [half rotation (HR)];
(c) the correct left–right turn requires a > 90°, < 180° mental rotation [full rotation (FR)].

Both the paper and the virtual Road Map present 8 NR, 16 HR, and 8 FR points.

Accordingly, a repeated measure 2x3x3 ANOVA was conducted. The statistic model includes as within factor Environment (2 levels: M-RMT/VR-MRT) ^∗^ Turn Type (3 levels: NR/HR/FR) ^∗^ Age Group (3 levels: YA/A/OA) as between factor.

Results showed a significant difference [*F*(648.83), *p* < 0.001] for Turn Type, for the interaction between Turn Type and Environment [*F*(179.53), *p* < 0.001] and for the interaction between Turn Type and Age Group. There was no statistical significance [*F*(2.34), *p* = 0.059] in the interaction between Turn Type, Environment, and Age Group.

With regards to the Environment, pairwise means comparison (*t*-test) revealed significant differences between M-RMT and VR-MRT for NR [*t*(60) = 17.86; *p* < 0.001], HR [*t*(60) = 18.69; *p* < 0.001], and FR [*t*(60) = 16.63; *p* < 0.001]. Performances in M-RMT revealed higher means compared to performances in VR-MRT.

With regards to the Turn Type, *post hoc* analysis with Bonferroni adjustment revealed significant differences between NR and HR (*p* < 0.001), HR and FR (*p* < 0.001), but no significant differences between NR and FR (*p* = 0.544) in the M-RMT. There are significant differences between NR and HR (*p* < 0.001), HR and FR (*p* < 0.001), and between NR and FR (*p* < 0.001) in the VR-RMT. Finally, significant differences between M-RMT and VR-RMT are observed for the three turn type(s).

At last, a *post hoc* analysis with Bonferroni adjustment revealed that Age Group influenced differently Turn Type performances in M-RMT and VR-RMT. For all the three types of rotations there are significant differences between YA and OA (NR *p* < 0.001; HR *p* < 0.001; FR *p* < 0.001), and between A and OA (NR *p* < 0.005; HR *p* < 0.003; FR *p* < 0.05) in M-MRT; while there are significant differences between YA and A (NR *p* < 0.001; HR *p* < 0.001; FR *p* < 0.001), and between A and OA (NR *p* < 0.001; HR *p* < 0.001; FR *p* < 0.001) in M-MRT. Detailed values are depicted in Table 2.

**Table 2:** Means and Standard deviations for rotation type, task and age group.

Age group	Environment	Rotation	Mean	*SD*
YA (form 30 to 49)	M-RMT	No rotation	7,421	0,223
		Half rotation	15,368	0,432
		Full rotation	7,632	0,233
	VR-RMT	No rotation	4,789	0,348
		Half rotation	9,684	0,677
		Full rotation	5,421	0,327
A (from 50 to 64)	M-RMT	No rotation	6,895	0,223
		Half rotation	13,895	0,432
		Full rotation	7,000	0,233
	VR-RMT	No rotation	2,053	0,348
		Half rotation	4,263	0,677
		Full rotation	2,632	0,327
OA (from 65 to 80)	M-RMT	No rotation	5,870	0,203
		Half rotation	11,609	0,393
		Full rotation	6,174	0,212
	VR-RMT	No rotation	1,391	0,316
		Half rotation	2,913	0,616
		Full rotation	2,130	0,297


## Conclusion

Starting from the enactive cognition approach the main research question proposed in this study was about the equivalence between a spatial orientation assessment obtainable from a classical neuropsychological test and the one obtainable from a virtual reality based one. Specifically, as in clinical neuropsychology the classical tests generally provide the patients with an allocentric simulation of space (e.g., a maze or a sketch map) the evaluation of spatial ability might differ from the one derived providing patients with the egocentric perspective possible in the virtual environments. In the classical assessment, in fact, an agent has to translate the allocentric perception in egocentred action, while during the virtual assessment the agent is allowed to move within the environment in the egocentric perspective. As the concept of enaction have introduced the notion of the coevolution of the agent and its environment, the main research question was about if it is possible to create equivalent representations of the surrounding environment in terms of opportunities for action (affordances) and sensorimotor invariants both in allocentred and egocentred spatial simulations.

In exploring a virtual environment an agent took embodied opportunities for action that are granted to the her from the simulation, on the basis of the atypical interaction provided by the computer simulated environment. These kind of affordances are not provided by the environment *per se* but from the interaction between the explorer and the virtual environment. Consequently, it appeared to be necessary to determine if the orientation obtainable from a virtual environment might differ from the spatial orientation obtainable from other kind of simulations (e.g., an analogical simulation like a sketched map). Thus, the different kind of body–environment coupling was analyzed here in two different forms of the same neuropsychological test.

Even if spatial cognition in virtual environment is comparable to the spatial orientation obtainable from the navigation other simulated spaces, due to the “sense of presence” experienced in it (Carassa et al., 2005; Riva et al., 2011), the present study revealed several significant differences between these two experimental conditions. The VR-RMT appears to be more complex to solve than the M-RMT. This difference between the two tests seems to be directly addressable to the complexity of the turn type in spatial exploration.

Considering nature of the tasks it is possible to observe that in the VR-RMT, the half of participants were asked to use the paper version of the test to perform turns in the virtual environment. It could have be interpreted as a dual task condition, requesting participants to first take a decision about the turns through using the paper-simulated environment and thus to translate the same decision in the virtual-simulated environment. To perform the VR-MRT requires a continuous attention focus change between the two simulations and a perspective switch between the survey of the M-RMT and the route of the VR-MRT. Thus, the finding that the performance was worse in this condition may not be very surprising.

Primarily it is possible to solve the M-RMT by imagining egocentric spatial transformations (Schultz, 1991) whereas in the VR-MRT, participants took decisions for each turn point being in front of the screen and by acting according to the appropriateness of their choices. The M-RMT and the VR-RMT differs in the imagined/perceived perspective taking because in the first task the agent have to set-out how to modify the turn on her body axis and how to derive a new perspective from that turn, whereas in the second task, the agent directly perform the turn on the body axis and directly perceive the point of view modification derived from it. Moreover, the VR-RMT does not require the participant to continuously re-locate herself looking at the map, because the track of each position is done by the experimenter and doesn’t require an additional cognitive effort.

Following the second interpretation, we expected a presentation order effect (between the group who experienced the M-RMT or the VR-RMT first) and also a better performance on the VR-RMT. Instead, the participants don’t express a presentation order effect and performed worse on the VR-RMT. Thus, independently from the presentation order, the VR-RMT was more complicated to perform than the M-RMT. A possible explanation of this experimental result may be related to the difference between simulation and action: rotating the body on its vertical axis toward the point of reference in virtual reality is more difficult than rotating the body in a mental space. Tversky (2009) underlines that human being continuously experience their own body from inside, influencing the peri-personal space that is independent from the physical environment *per se*. Moreover, it is possible to consider perspective taking and mental rotation as dissociated. When perspective taking, in fact, includes thinking about the changing of the owns egocentric perspective with respect to the surroundings, the mental rotation includes thinking about the effects of modifying the placements of objects in the surroundings during the maintenance of owns actual perspective in the environment (Hegarty and Waller, 2004).

In addiction, Hintzman et al. (1981) describe spatial knowledge as derived from orientation-specific perspectives, and of relational propositions. Accordingly Kozhevnikov and Hegarty (2001) indicate as the main strategy used in understanding a more than 90° perspective task is to imagine oneself reoriented with respect to the scene. This strategy could have to be used from this study participants. For both the M-RMT and VR-RMT, in order to follow the route participants have to imaginatively anticipate themselves in specific orientation. Generally, an agent is able to move on the gravitational axis while the environment doesn’t provide variations. This kind of embodied turn creates an expectation about the spatial perspective (defined by Gibson’s affordance theory as “invariants of the physical world”) that could have been more efficacious in updating an imaginative world compared to the one of the virtual environment.

These results appear to be partially incongruent with current research in the field. As introduced by Gray and Fu (2004), in interacting with computer-based simulation, individuals were given the option of using the external visualizations to perceive the effect of their actions rather than relying on internal visualization to imagine the effect. In accord with Keehner et al. (2008), it is possible to think that in the VR-MRT task the agents matched the virtual environment snapshots with the right/left turn intentions in looking for the match between the obtained perspectives and the effect of each turns. This continuous reference matching can be considered as tightly coupled with internal cognitive processes. The possibility to externalize representations provided by VR-RMT (by observing the perspective resulting from a right/left turn) may have required more effort than to base it on the embodied imaginative process (as in the M-RMT). This data interpretation is also consistent with the perspective proposed by Di Paolo (2005). Accordingly, here we can suggest that in the VR-MRT, a failure of the sensorimotor coupling between the perceptual information and the turn response on the virtual scenario that doesn’t involve the entire body, might have created a meaningless experience for the agent during navigation. Thus the failure of the sensorimotor coupling has been considered as quite useless for spatial orientation.

It is also possible to mention that in the VR-RMT, each mistake in turn taking provides a difference between the agents’s expected and taken perspective in space that might influence the next turns affecting the final result more in the VR-RMT than in the M-RMT. This interpretation of the data appears to be supported by the analysis of our results on turn type. Managing HR/FR appears to be easier in the imaginative task than in the virtual one. This is largely observable from the individual differences in the analysis of our data: the results from Age Group comparison showed that our participants were not all equally able to use external visualizations to support spatial orientation in virtual reality. Moreover, the ability to orient them VR-RMT decreases with age. The interaction between Environment and Age Group, in fact, revealed how there is a difference between the younger groups (YA and A) and the older population wayfinding performed in the M-RMT. It reveals a decline with age in the allocentric to egocentric spatial translation. Whereas in VR-MRT there is a difference of the YA both when compared to A and OA.

This result confirms that the orientation task both in M-RMT and VR-RMT is not equal for all individuals but that it is strictly dependent on the participants’ age. Moreover, our data appear to be consistent with the recent findings in age-related decline for wayfinding in complex environments Harris and Wolbers (2014). By using a complex virtual environment for wayfinding ability evaluation in young and old populations they found a wide role of age on the capacity to change from route knowledge to survey one in order to find a target location. Moreover, in their work older participants showed evidence of difficulties in route to survey switching performance, confirming that it can be at least partly explained in terms of prefrontal-noradrenergic network impairment, responsible for egocentric to allocentric coordinating switching behavior.

Finally, the interaction between age group and spatial performances could be also addressed to a computer expertise that can be derived from the age of our participants. We have assumed, in fact, that our age cohorts reflect the possible everyday use of computer or other technological devices in the participants’ everyday lives. We had the YA group that could be defined as a “digital native” and were largely exposed to computer-based interactions, the A group that is still a working population and could be quite expert in computer use, and the Old Adult group that is probably retired from work and might not have a large expertise with technologies. These groups appear to be different between M-RMT and VR-RMT. In VR-RMT it appears clear how OA had difficulties in managing turns and that it could be related to the participants’ expertise in using computer-based simulations. The data derived from VR-RMT condition are consistent with the evidence that a variability between subjects in spatial task performance is high in virtual reality spaces (Klatzy et al., 1998; Waller et al., 1998). Most of the cognitive abilities involved in understanding space in a virtual simulation seems to be higher cost demanding.

As described above, by considering the sync between both the perspective as essential for spatial navigation and wayfinding, the differences in spatial evaluation obtainable from mainly allocentric or mainly egocentric environment simulations (and from the possibility of interaction they differently provide) have been deeply investigated. Consequently, in order to obtain solid data seems to be necessary to think about an assessment tool specific for virtual environment application (Belingard and Péruch, 2000; Waller, 2000, 2005). Otherwise, within the enactive perspective on cognition, data derived from spatial tasks performed through virtual reality simulations in largely restrictive action possibilities (e.g., neuroimaging studies) could be considered as not completely reliable.

As cognition is the form of embodied action in which cognitive processes arise from recurrent sensorimotor patterns of perception and action (Thompson, 2005), the coupling between organism and environment modulates the construction of a relational domain that is not internally represented in the brain but it is created from the activity and the peculiar coupling with the specific environment. This evidence suggests that the opportunity of including virtual environments in cognitive evaluation is not exclusively technological, but epistemic. Thus, for spatial cognition evaluation, beyond considering the virtual simulation appropriateness, is equally important to understand the enaction stance that acknowledges orientation as derived from egocentric/allocentric sensorimotor invariance. Data presented here revealed how this sensorimotor invariance differed from the possibility of offloading spatial knowledge, as in the classical and virtual version of M-RMT.

Hence, enactive cognition can be considered ad a privileged point of view in examining virtual reality as more than purely digital place, but as a technical challenge in which an agent is able to find spatial invariants, and to progressively evolve them through the dynamics of the sensory-motor coupling. In this way she understand the environment and the possibilities for action in it.

Thus, the introduction on virtual reality in cognitive science research have to consider how this kind of simulation more than being “realistic” has to technically support the agents’ possibility to potentially distinguish the moment-by-moment different paths of encounters with the environment (Di Paolo, 2005, 2009). The peculiar possibilities of sensorimotor coupling, defined for example from the environment characteristics and from the interaction design possibilities provided to the agent can supply explorers with “virtual reality- based” affordances for action and differentiated information feedbacks. Each of these should be deeply considered in order to understand how they could provide distinctive effort for spatial knowledge.

At last, the inclusion of virtual environments within the assessment tools for spatial cognition in neuropsychology may provide an interesting alternative to paper and pencil-based approaches but data derived from this evaluation have to be used with extremely caution. Virtual environments in fact appear here to not involve the same embodied spatial information derived from the navigation performed in other types of environments. Even if it remains a great challenge for enactive cognition research (Varela, 1990).

## Ethics Statement

This study was carried out in accordance with the recommendations of University of Bergamo with written informed consent from all subjects. All subjects gave written informed consent in accordance with the Declaration of Helsinki. The protocol was approved by the University of Bergamo research office.

## Author Contributions

FM ideated the experiment, collected and analyzed the data, wrote the manuscript.

## Conflict of Interest Statement

The author declares that the research was conducted in the absence of any commercial or financial relationships that could be construed as a potential conflict of interest.
